# TrkA inhibition alleviates bladder overactivity in cyclophosphamide-induced cystitis by targeting hyperpolarization-activated cyclic nucleotide-gated channels

**DOI:** 10.22038/IJBMS.2023.68528.14943

**Published:** 2023

**Authors:** Qian Liu, Xiaodong Li, Jingzhen Zhu, Bishao Sun, Shadan Li

**Affiliations:** 1 Clinical Medicine Postdoctoral Research Station, The First Affiliated Hospital of Chongqing Medical University, Chongqing, China; 2 Department of Urology, The General Hospital of Western Theater Command, Chengdu, China; 3 Department of Urology, The Affiliated Hospital of Southwest Medical University, Luzhou, China; 4 Department of Urology, Second Affiliated Hospital, Army Medical University, Chongqing, China

**Keywords:** Cyclophosphamide, Cystitis, Hyperpolarization-activated-cyclic nucleotide-gated channels, Tropomyosin receptor-kinase A, Urinary bladder

## Abstract

**Objective(s):**

To investigate the potential of Tropomyosin receptor kinase A (TrkA) for the treatment of interstitial cystitis/ bladder pain syndrome (IC/BPS).

**Materials and Methods:**

Sixty-four female rats were randomly assigned to the control and cyclophosphamide (CYP) groups. Quantitative reverse transcription polymerase chain reaction was utilized to detect the mRNA level of TrkA. Western blot analysis was used to measure the protein levels of TNF-α, IL-6, and TrkA. Immunostaining was used to detect the expression of TrkA in bladder sections. Contractility studies and urodynamic measurements were utilized to test the spontaneous contractions of detrusor muscle strips and the global bladder activity, respectively.

**Results:**

Rat models of chronic cystitis were successfully established. The mRNA and protein levels of TrkA were significantly increased in the bladders of CYP-treated rats. Also, results of immunohistochemical staining and immunofluorescence staining showed that increased TrkA expression in the CYP group was mainly observed in the urothelium layer and bladder interstitial Cajal-like cells (ICC-LCs) but not in the detrusor smooth muscle cells. The specific inhibitor of TrkA, GW441756 (10 μM), significantly suppressed the robust spontaneous contractions of detrusor muscle strips in the CYP group and alleviated the overall bladder overactivity of CYP-treated rats. However, the inhibitory effects of GW441756 (10 μM) on the spontaneous contractions of detrusor muscle strips and the overall bladder activity were eliminated after pretreatments with the specific blocker of hyperpolarization-activated cyclic nucleotide-gated (HCN) channels, ZD7288 (50 μM).

**Conclusion:**

Our results suggested that increased TrkA expression during chronic cystitis promotes the development of bladder overactivity by targeting the HCN channels.

## Introduction

Interstitial cystitis/ bladder pain syndrome (IC/BPS) is a chronic bladder disorder characterized by a series of symptoms, such as urinary frequency, urgency, nocturia, and pelvic pain (1). In the United States, the prevalence of IC/BPS is estimated to be 2.7% to 6.53% in women (2). Furthermore, IC/BPS is more prevalent in women with an approximate female-to-male ratio ranging from 5:1 to 10:1 (3). IC/BPS negatively impacts the health-related quality of life, resulting in damaged work life and an increased risk of depression and suicidal ideation (4, 5). To date, the etiology of IC/BPS remains unclear and various hypotheses have been proposed, such as deficiency of the glycosaminoglycan layer, enhanced urothelial permeability, mast cell activation, and neurogenic inflammation, etc. (6). However, current treatments against IC/BPS based on these hypotheses are still insufficiently effective. Therefore, the pathogenesis of IC/BPS needs to be further explored to develop more therapeutic strategies.

Nerve growth factor (NGF), a member of the neurotrophin family, occupies a crucial role in regulating the growth, differentiation, and survival of neurons in the central and peripheral nervous system (7). NGF exerts its biological effects by binding to two classes of receptors: the specific high-affinity tropomyosin receptor kinase A (TrkA) and the common low-affinity p75 neurotrophin receptor (p75NTR) (8). Accumulated evidence has demonstrated that aberrant NGF/TrkA signaling is associated with many disorders, such as intractable pain, Alzheimer’s disease, and diverse cancers (9-11). In the bladder, NGF is produced by the urothelium, detrusor smooth muscle cells, and mast cells upon stretch or inflammation (12). Clinical research data have shown that urinary NGF levels are raised in multiple hyperactive bladder dysfunctions, such as IC/BPS and overactive bladder (13, 14). A clinical trial has also revealed that NGF is an effective therapeutic target for IC/BPS (15). Besides, previous studies reported that TrkA expression was significantly increased in bladder tissues of rodent models with cyclophosphamide (CYP)-induced cystitis (16, 17). However, the potential of TrkA for the treatment of IC/BPS has not been investigated. More studies based on TrkA inhibitors may provide more novel therapeutic approaches for IC/BPS.

Hyperpolarization-activated cyclic nucleotide-gated (HCN) channels belong to the voltage-gated pore loop channel superfamily with four subtypes (HCN1-4) existing in mammals (18). HCN channels have been found to mediate the pacemaker current I_h_ and contribute to neuronal and cardiac rhythmic activity (19). HCN channels are also important modulators of the spontaneous activity of the bladder (20). Our previous study has revealed the crucial role of HCN channels in inducing bladder hyperactivity during CYP-induced cystitis (21). Besides, it has been reported that HCN channels are involved in the regulation of neuronal excitability by the 5-hydroxytryptamine 1A receptor or cannabinoid type-1 receptors (22, 23). Furthermore, our previous study has demonstrated that activation of the prostaglandin EP3 receptor could enhance bladder excitability by increasing the expression and function of HCN channels (24). Based on the aforementioned findings, we supposed that NGF/TrkA signaling may also promote cystitis-associated bladder overactivity through modulating HCN channels. In the present study, we detected that TrkA expression in the bladder was significantly increased in the rat model of CYP-induced chronic cystitis, and demonstrated that TrkA inhibition by its selective inhibitor GW441756 significantly relieved chronic cystitis-associated bladder overactivity through targeting HCN channels.

## Materials and Methods

***Animals***

Sixty-four female Sprague-Dawley rats (200-220 g) were purchased from the Experimental Animals Center of Army Medical University (Chongqing, China) and randomly assigned to the control and CYP groups. All rats were housed in cages under the conditions of a temperature of 23±1 ^°^C and a 12 hr light/dark cycle. All rats were also given *ad libitum* access to water and food. Animal experiments in the present study were approved by the Research Council and Animal Care and Use Committee of the Army Medical University and were performed in accordance with the Guide for the Care and Use of Laboratory Animals published by the National Institutes of Health.

***Animal model of chronic cystitis***

Rat models of chronic cystitis were induced using CYP as previously described (25, 26). Briefly, rats in the CYP group and control group received intraperitoneal injections of CYP (75 mg/kg, Sigma, St Louis, MO, USA) and equal volumes of sterile saline on days 1, 4, and 7, respectively. All rats were sacrificed for subsequent experiments 24 hr after the last injection. 

***Hematoxylin-eosin (HE) staining***

HE staining was performed to detect the histological changes in bladder sections. After fixation with 4% paraformaldehyde, rat bladders were dehydrated and embedded in paraffin, and sliced into 4 μm-thickness sections. Thereafter, sections were stained with hematoxylin for 5 min and eosin for 2 min, followed by sealing with neutral gum. All sections were photographed using an upright optical microscope (Olympus, Tokyo, Japan).

***Quantitative reverse transcription polymerase chain reaction (RT-PCR)***

Quantitative RT-PCR was performed to detect the mRNA expression level of TrkA. Total RNA in the rat bladder was isolated by the Trizol reagent (Beyotime, Shanghai, China) and subsequently synthesized to cDNA utilizing a PrimeScript™ RT reagent kit (Takara, Kusatsu, Shiga, Japan). Primer sequences were listed in Table 1. Quantitative RT-PCR was run in a StepOnePlus Real-Time PCR system (Applied Biosystems, Foster City, CA, USA) with the SYBR Green Mix (Toyobo, Osaka, Japan) for 40 cycles. Relative TrkA expression normalized to GAPDH expression was analyzed using the 2^-ΔΔCt^ method.

***Western blot analysis***

Western blot analysis was conducted to measure the protein expression levels of TNF-α, IL-6, and TrkA. The total protein of the rat bladder was extracted by RIPA lysis buffer (Beyotime, Shanghai, China) supplemented with 1% PMSF (Beyotime), and its concentration was measured using a BCA kit (Beyotime). Equal amounts of protein samples were electrophoretically separated by the SDS-PAGE gel and then transferred onto the PVDF membrane (Merck Millipore Ltd., Tullagreen, Carrigtwohill, Co. Cork, Ireland). After being blocked in 5% skim milk at room temperature for 2 hr, the membranes were incubated overnight at 4 ^°^C with the following primary antibodies: anti-TNF-α (Abcam, Cambridge, MA, USA, ab199013, 1:500), anti-IL-6 (Abcam, ab9324, 1:800), anti-GAPDH (Beyotime, AG019, 1:1000), anti-TrkA (CST, Inc., Danvers, MA, USA, #2505, 1:500), anti-α-tubulin (Beyotime, AT819, 1:1000), followed by incubation with horseradish peroxidase (HRP)-conjugated goat anti-rabbit IgG (Invitrogen, Carlsbad, California, USA, G21234, 1:5000) or goat anti-mouse IgG (Invitrogen, G21040, 1:5000) at room temperature for 2 hr. At last, the membranes underwent chemiluminescence with the Pierce™ ECL Western substrate (Thermo Fisher Scientific, Waltham, MA, USA) in an Azure Biosystems C300. 

***Immunohistochemical staining***

Immunohistochemical staining was conducted to detect the expression of TrkA in bladder sections. Paraffin-embedded bladder sections were orderly dewaxed and hydrated, and immersed in 0.01 M citrate buffer (pH 6.0) at 95 ^°^C for 5 min to restore the antigen. After being blocked with goat serum for 1 hr, sections were incubated with the primary antibody of anti-TrkA (CST, #2505, 1:100) at 4 ^°^C overnight, followed by incubations with biotin-labeled goat anti-rabbit IgG (Bioss, 1:300) and streptavidin-biotin complex kit (Beyotime) at 37 ^°^C for 30 min, respectively. Each incubation step was followed by PBS washes three times. Sections were then stained with diaminobenzidine (Beyotime) and hematoxylin and photographed using an upright optical microscope (Olympus). 

***Immunofluorescence staining***

Immunofluorescence staining was performed to detect the expression of TrkA on c-kit-positive cells in bladder sections. Rat bladders were cut into 6 μm-thickness sections in a freezing microtome and were immediately fixed with 4% paraformaldehyde for 20 min. Sections were then blocked with immunostaining blocking buffer (Beyotime) at room temperature for 1 hr. After incubation at 4 ^°^C overnight with primary antibodies of anti-C-kit (Santa Cruz, Dallas, TX, USA, sc-365504, 1:50) and anti-TrkA (CST, #2505, 1:100), sections were subsequently incubated in the dark with Alexa 488-conjugated goat anti-mouse IgG and Alexa 647-conjugated donkey anti-rabbit IgG (Bioss, Beijing, China, 1:200) at room temperature for 90 min, followed by incubation with DAPI (Beyotime) for 10 min. Each incubation step was followed by PBS washes three times. Sections were finally photographed under a laser-scanning confocal microscope (Leica, Wetzlar, Germany). 

***Contractility studies***

Contractility studies were conducted to test the spontaneous contractions of detrusor muscle strips. The compounds of Kreb’s solution were as follows (in mM): 119 NaCl, 4.7 KCl, 1.2 KH_2_PO_4_, 1.2 MgSO_4_•7H_2_O, 25 NaHCO_3_, 2.5 CaCl_2_, and 11 glucose; adjusted to pH 7.4. Rat bladders were cut into longitudinal detrusor muscle strips (3×4×8 mm). Each strip was vertically placed in 15 ml of Kreb’s solution and maintained with a gas mixture of 95% O_2_ and 5% CO_2_ at 37 ^°^C*.* One end of each strip was linked with the RM6240C signal processing system (Chengyi Co., Chengdu, China) via a tension transducer, and the other end was fixed to the bottom of the organ bath. Each strip was applied a basic tension of 0.75 g after equilibration for 30 min, and its spontaneous contractions were recorded in the RM6240C system. When the muscle strips were steadily contracted, the selective inhibitor of TrkA (GW441756, 10 *μM*, Tocris Bioscience, Bristol, UK) or the vehicle (DMSO) was added into Kreb’s solution. In addition, the selective blocker of HCN channel (ZD7288, 50 *μM*, Sigma, St Louis, MO, USA) and 10 *μM* GW441756 were sequentially added into the Kreb’s solution at a 6-min intervals.

***Urodynamic measurements***

The global bladder activity of rats was tested using urodynamic measurements. Rats were anesthetized by intraperitoneal injection of urethane (1 g/kg body weight, Sigma) prior to receiving a fistulation in the bladder dome. The bladder incision was then closed using a purse-string suture after inserting a PE-50 polyethylene catheter (Becton Dickinson & Company, Franklin Lakes, New Jersey, USA) into the bladder. The PE-50 catheter was linked to a three-way valve, which was connected to an infusion pump (Minnesota Mining and Manufacturing Co., Saint Paul, MN, USA) and a pressure transducer. Room temperature saline dissolved with GW441756 (10 μM) or DMSO was constantly infused (10 ml/hr) into the bladder. Additionally, saline containing ZD7288 (50 μM) with or without GW441756 (10 μM) was also orderly infused into the bladder. Continuous urodynamic curves were recorded in the RM6240C system (Chengyi Co.). 

***Statistical analysis***

All data are represented as mean±SD. Statistical analyses were performed with the methods of independent samples t-test and two-way analysis of variance (ANOVA), using the SPSS version 16.0 (SPSS Inc., Chicago, IL, USA) and GraphPad Prism version 8.0 (San Diego, CA, USA), respectively. A *P*-value<0.05 was considered statistically significant.

## Results

***Successful modeling of CYP-induced chronic cystitis in rats***

**Figure 1:**
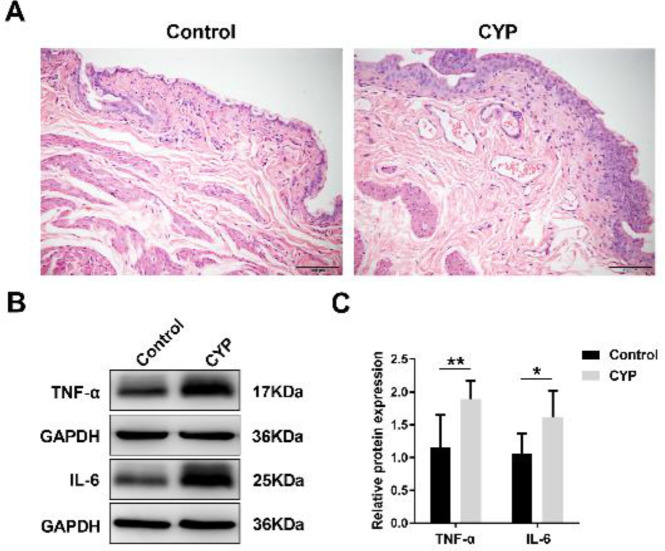
Successful modeling of CYP-induced chronic cystitis in rats

We first assessed whether the rat models of CYP-induced chronic cystitis were successfully established. The results of H&E staining demonstrated that the rat bladder section in the CYP group showed moderate hyperemia and edema in the lamina propria together with urothelial hyperplasia, compared with that in the control group (Figure 1A). Moreover, we detected that the protein expression levels of two inflammatory cytokines namely TNF-α and IL-6 in the rat bladder of the CYP group were significantly increased compared with those of the control group (Figure 1B and C). The above results confirmed the successful modeling of CYP-induced chronic cystitis in rats. 

***TrkA expression was significantly increased in the bladder of CYP-treated rats***

**Figure 2:**
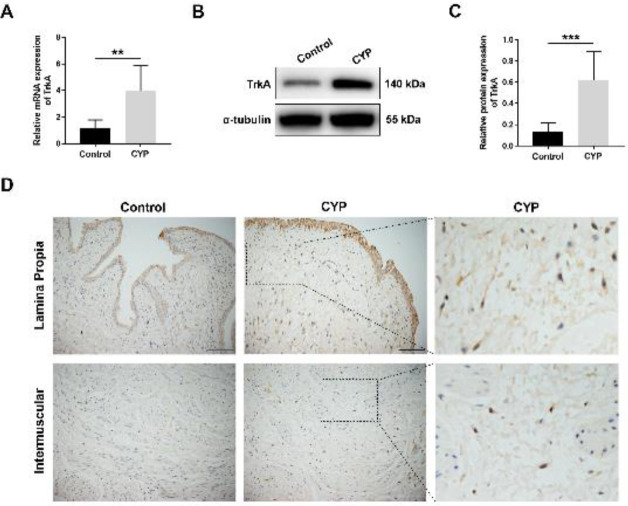
Tropomyosin receptor kinase A (TrkA) expression was significantly increased in the bladder of cyclophosphamide (CYP)treated rats

Next, we found that the mRNA and protein levels of TrkA in rat bladders of the CYP group were significantly elevated compared with those of the control group (Figure 2A-C). As shown in Figure 2D, the results of immunohistochemical staining showed that TrkA expression in the bladder section of the CYP group was obviously enhanced in the urothelium, whereas not in the detrusor smooth muscle cells. Furthermore, TrkA expression in interstitial cells located in the lamina propria and intermuscular region of the CYP group was also observed to be remarkably increased. 

**Figure 3:**
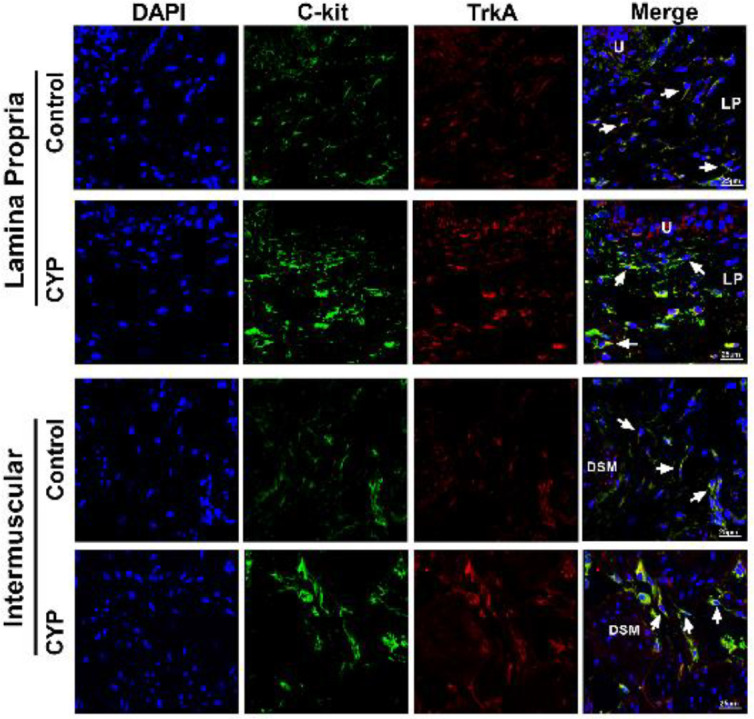
Tropomyosin receptor kinase A (TrkA) expression in bladder ICC-LCs was prominently increased in cyclophosphamide (CYP) treated rats

To further test whether TrkA expression was changed in an important type of interstitial cells in the bladder, interstitial Cajal-like cells (ICC-LCs), we performed double-labeling immunofluorescence staining. In the lamina propria and intermuscular region of bladder sections, we observed that TrkA was co-labeled with the C-kit (an acknowledged marker of bladder ICC-LCs) in some stellate or elongated-shaped cells, indicating that TrkA was expressed in bladder ICC-LCs. We found that the TrkA expression level in bladder ICC-LCs of the CYP group was significantly higher than that of the control group. In addition, the results of immunofluorescence staining further confirmed that TrkA expression in the urothelium, rather than in the detrusor smooth muscle cells, was obviously increased in the CYP group compared with the control group (Figure 3). 

***TrkA inhibition by GW441756 significantly ameliorated bladder overactivity of CYP-treated rats***

**Figure 4:**
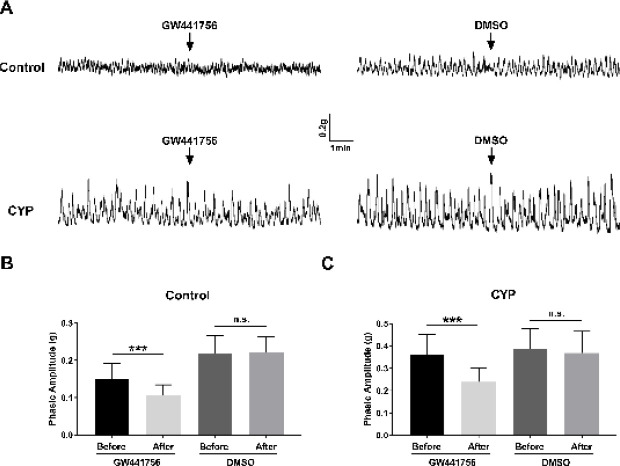
Tropomyosin receptor kinase A (TrkA) inhibition by GW441756 significantly suppressed the spontaneous contractions of detrusor muscle strips isolated from control and cyclophosphamide (CYP) treated rats

We further tried to explore the potential role of TrkA in cystitis-associated bladder overactivity. To this end, we first applied a specific inhibitor of TrkA, GW441756, to test its effect on bladder contractility. In contractility studies, we found that the spontaneous contractions of the detrusor muscle strip isolated from the CYP-treated rat were more robust than those of the control rat. Moreover, we detected that GW441756 (10 μM), rather than the vehicle DMSO, significantly suppressed the spontaneous contractions of detrusor muscle strips in both the control and CYP groups (Figure 4A). Upon the treatment of GW441756, the reduction in phasic amplitude of spontaneous contractions of detrusor muscle strips was approximately 30% and 35% in the control group and the CYP group, respectively (Figure 4B and C). 

**Figure 5:**
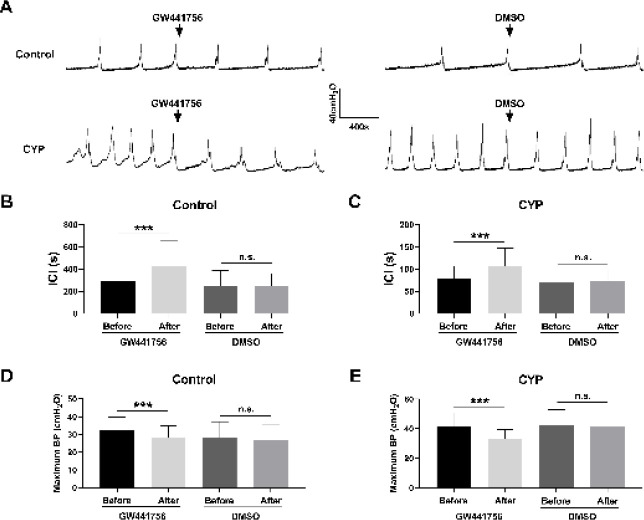
GW441756 significantly ameliorated the overall bladder overactivity of cyclophosphamide (CYP) treated rats

Next, we tested the impact of GW441756 on the overall bladder activity using urodynamic measurements. The results showed that CYP-treated rats exhibited apparent bladder overactivity compared with control rats, which was characterized by a striking decrease in the intercontractile interval (ICI) and a prominent increase in the maximum bladder pressure (MBP) (Figure 5A). Upon the administration of GW441756 (10 μM), the ICI and MBP of both the control and CYP-treated rats were significantly extended and reduced, respectively. On the contrary, the DMSO had no significant effect on such urodynamic parameters of control and CYP-treated rats (Figure 5B-E). Taken together, the above findings indicated that GW441756 significantly ameliorated bladder overactivity of CYP-treated rats. 

***HCN channels participated in the modulation of bladder activity by TrkA***

**Figure 6:**
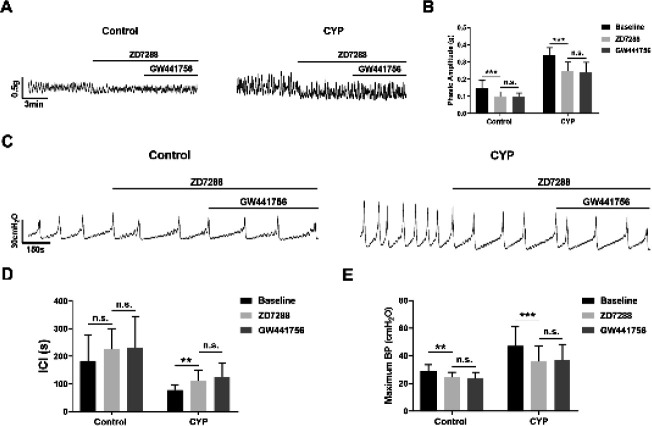
hyperpolarization-activated cyclic nucleotide-gated (HCN) channels participated in the modulation of bladder activity by Tropomyosin receptor kinase A (TrkA)

Finally, we attempted to investigate whether HCN channels were involved in the regulation of bladder activity by TrkA. In contractility studies, we found that the inhibitory effects of GW441756 (10 μM) on the spontaneous contractions of detrusor muscle strips in both the control and CYP groups were eliminated after pre-incubation with the specific blocker of HCN channels, ZD7288 (50 μM) (Figure 6A and B). Furthermore, similar results were obtained in urodynamic measurements. Upon administration of ZD7288 (50 μM), GW441756 (10 μM) no longer significantly prolonged the ICI and decreased the MBP of control and CYP-treated rats (Figure 6C-E). These results indicated that HCN channels may participate in the regulation of bladder activity by TrkA.

## Discussion

Upon systematic administration, CYP can be metabolized to acrolein, which subsequently accumulates in the bladder and ultimately leads to hemorrhagic cystitis (27). An experimental rodent model induced by chronic low-dose administration of CYP is widely used to mimic the clinical features of IC/BPS (28, 29). Accordingly, we also established the rat models of CYP-induced chronic cystitis in our present study. To date, abundant clinical and experimental evidence has demonstrated that NGF production is significantly increased in the bladder of IC/BPS patients and CYP-treated rats (30, 31). Therefore, we only detected whether TrkA expression was altered in the bladder of our rat models. A previous study has reported that TrkA expression was significantly increased in bladder specimens of patients with severe ketamine‐associated cystitis (32). Our results also demonstrated that the overall expression of TrkA in the rat bladder was significantly increased after CYP treatments for 8 days. These results indicate the potentially important role of TrkA during the development of cystitis. Furthermore, our findings were consistent with that reported by another previous study in which the protein expression of TrkA was found to be significantly enhanced in the bladder of Wistar rats with CYP-induced intermediate (48 hr) or chronic (10 days) cystitis (17). Besides, this previous study also found that the increase in TrkA expression upon chronic CYP treatments was mainly observed in detrusor muscle cells using immunofluorescent staining (17). However, our findings in immunohistochemical staining revealed that CYP-induced increase in TrkA expression was obviously observed in the urothelium and interstitial cells, but not in detrusor muscle cells. In addition, our results of immunofluorescent staining also did not show an obvious increase in TrkA expression in detrusor muscle cells of CYP-treated rats compared with control rats. We speculate that this inconsistency may be attributed to the discrepancies between animals or the different modeling methods. 

Nowadays, increasing evidence has revealed the crucial role of the urothelium in the micturition reflex. Firstly, the urothelium possesses specialized sensory properties due to its expressed receptors and ion channels. Moreover, the urothelium can also exert its role of signal transduction by functionally interacting with nerves, detrusor smooth muscle cells, and bladder ICC-LCs (33). Besides, bladder ICC-LCs are considered possible electrical pacemakers to mediate bladder activity (34). Changes in the urothelium and bladder ICC-LCs are closely related to the hypersensitivity of overactive bladder (35). In the present study, we further confirmed that chronic CYP treatments prominently enhanced TrkA expression in the urothelium and bladder ICC-LCs using double-labeling immunofluorescence. Furthermore, our results of contractility studies demonstrated that the specific TrkA inhibitor GW441756 significantly suppressed the spontaneous contractions of detrusor muscle strips isolated from control and CYP-treated rats. Above findings suggest that NGF/TrkA signaling is implicated in the regulation of bladder activity, even under the condition of denervation. Increased TrkA expression in the urothelium and bladder ICC-LCs may partially contribute to chronic cystitis-associated bladder overactivity.

In recent years, several anti-NGF monoclonal antibodies have been developed for the treatment of IC/BPS, such as tanezumab and fulranumab (36). A clinical trial demonstrated that tanezumab significantly relieved pain and decreased the frequency of urgency episodes, but had no significant effect on micturition frequency in patients with IC/BPS (15). As we know, except for its specific high-affinity receptor TrkA, NGF also can exert its biological effects via binding to its low-affinity receptor p75NTR (8). The expression level of p75NTR was significantly elevated in the rat bladder with CYP-induced cystitis, and blockade of NGF/p75NTR signaling by intravesical instillation of PD90780 induced and aggravated bladder hyperreflexia in control and CYP-treated rats, respectively (37). These results indicate that NGF/p75NTR signaling may play a role in maintaining the stability of bladder activity under physiological and inflammatory conditions. Therefore, we suggest that specific inhibition of NGF/TrkA signaling may be a more ideal treatment strategy for IC/BPS. A previous study demonstrated that oral medication of GW441756 significantly reduced bladder hyperactivity and elevated the threshold of mechanical pain in rat models of chronic stress (38). In our present study, the results of urodynamic measurements demonstrated that intravesical perfusion of GW441756 significantly ameliorated bladder overactivity in rats with CYP-induced chronic cystitis. The above results suggest that TrkA may be a novel and effective target for the treatment of IC/BPS. Furthermore, owing to the diverse biological effects of NGF, systematic administration of anti-NGF monoclonal antibody in patients commonly faces various adverse events, such as abnormal peripheral sensation or the progression of osteoarthritis or osteonecrosis (36). Based on our results, we think that intravesical perfusion is a viable administration route for the inhibitor of NGF/TrkA signaling in the treatment of IC/BPS to avoid systematic adverse effects. 

NGF binding to TrkA can activate several downstream signaling pathways including phosphatidylinositol 3-kinase (PI3K), mitogen-activated protein kinase (MAPK), and phospholipase C-γ (PLC-γ) pathways (12). A previous study demonstrated that NGF significantly up-regulated the protein expression of transient receptor potential V1 (TRPV1) in dorsal root ganglia (DRG) neurons through the PI3K/protein kinase B (AKT) pathway (39). Another study described that NGF/TrkA axis significantly increased the expression and phosphorylation level of TRPV1 in DRG neurons by activating A-kinase anchoring protein 5, a scaffolding protein for protein kinase C (PKC) (8). Furthermore, TRPV1 was proven to be involved in NGF-induced detrusor overactivity (40). In addition, NGF/TrkA signaling also increased the expression of transient receptor potential melastatin 8 through the actions of PI3K, MAPK, c-Jun N-terminal kinase, and Src tyrosine kinase (41). The aforementioned findings indicate that NGF/TrkA signaling can regulate the expression and function of diverse ion channels via its numerous downstream protein kinases. Our previous research demonstrated that increased HCN channels were essential for cystitis-associated bladder hyperactivity (21). It is well-known that HCN channels can be phosphorylated and activated by various protein kinases, such as p38-MAPK, PKC, calcium/calmodulin-dependent protein kinase II (CaMKII), etc (18). Besides, evidence has shown that NGF binding to TrkA significantly facilitated the gating of the HCN2 channel through PLCγ activation in *Xenopus* oocytes (42). Accordingly, we speculate that HCN channels may be involved in the regulation of bladder activity by NGF/TrkA signaling. In our further contractility studies and urodynamic measurements, we found that pre-blockade of HCN channels by ZD7288 significantly eliminated the inhibitory effects of GW441756 on spontaneous contractions of detrusor muscle strips and global bladder activity in control and CYP-treated rats. These results preliminarily verify our speculation and also suggest that the improvement of cystitis-associated bladder overactivity induced by TrkA inhibition could be achieved by targeting HCN channels. The limitation of our present study was that we did not further investigate which downstream signaling pathway of the NGF/TrkA axis is implicated in its modulation of HCN channels. Further studies are required to be carried out to clarify the exact mechanisms underlying the regulation of HCN channels by NGF/TrkA signaling during cystitis. 

**Table 1:** Sequences of primers used for RT-PCR

**Gene**	**Species**	**Primer sequences**
TrkA	Rat	F: 5’-GAGTTGAGAAGCCTAACCATCG-3’ R: 5’-AAGCATTGGAGGAGAGATTCAG-3’
GAPDH	Rat	F: 5’-GGCCCCTCTGGAAAGCTGTG-3’ R: 5’-CCAGGCGGCATGTCAGATC-3’

TrkA: tropomyosin receptor kinase A; GAPDH: glyceraldehyde phosphate dehydrogenase; F: forward; R: reverse

(A) Hematoxylin-eosin staining of bladder sections from control and CYP-treated rats. Magnification: 200X. Scale bar: 100 μm. (B) Western blot analyses of TNF-α, IL-6, and GAPDH in the bladder of control and CYP-treated rats. (C) Relative protein expression levels of TNF-α and IL-6 normalized to GAPDH (n=3, *P<0.05 and **P<0.01, independent samples t-test)

(A) Relative mRNA expression level of TrkA normalized to GAPDH in the bladder of control and CYP-treated rats (n=6, **P<0.01, independent samples t-test). (B) Western blot analyses of TrkA and α-tubulin in the bladder of control and CYP-treated rats. (C) Relative protein expression level of TrkA normalized to α-tubulin (n=6, ***P<0.001, independent samples t-test). (D) Immunohistochemical staining of TrkA in bladder sections from control and CYP-treated rats. The lamina propria and intermuscular region in the bladder section of each group are displayed. Magnification: 200X. Scale bar: 100 μm

Immunofluorescence staining of TrkA (red) in bladder sections from control and CYP-treated rats. Bladder ICC-LCs (white arrows) located in the lamina propria and intermuscular region were labeled by C-kit (green). DAPI (blue) was utilized to label cell nuclei. DSM, detrusor smooth muscle; LP, lamina propria; U, urothelium layer. Scale bar: 25 μm

(A) Contractility studies of detrusor muscle strips isolated from control and CYP-treated rats upon the treatments of GW441756 (10 μM, the specific inhibitor of TrkA) and DMSO (Vehicle). (B, C) Statistical analyses of the phasic amplitude of spontaneous contractions of detrusor muscle strips isolated from control and CYP-treated rats (n=6, ***P<0.001 and n.s., no significance, two-way ANOVA)

(A) Urodynamic measurements of control and CYP-treated rats upon the treatments of GW441756 (10 μM) and DMSO (Vehicle). (B-E) Statistical analyses of the ICI and MBP (n=6, ***P<0.001, and n.s., no significance, two-way ANOVA)

(A) Contractility studies of detrusor muscle strips isolated from control and CYP-treated rats upon the sequential administrations of ZD7288 (50 μM, the specific blocker of HCN channels) and GW441756 (10 μM). (B) Statistical analyses of the phasic amplitude of spontaneous contractions of detrusor muscle strips isolated from control and CYP-treated rats (n=6, ***P<0.001, n.s., no significance, two-way ANOVA). (C) Urodynamic measurements of control and CYP-treated rats upon the sequential treatments of ZD7288 (50 μM) and GW441756 (10 μM). (D, E) Statistical analyses of ICI and MBP (n=6, **P<0.01, ***P<0.001, and n.s., no significance, two-way ANOVA)

## Conclusion

The present study demonstrated that the expression of TrkA was significantly increased in the urothelium and bladder ICC-LCs of rats with CYP-induced chronic cystitis, and TrkA inhibition significantly alleviated chronic cystitis-associated bladder overactivity by targeting the HCN channels. Our findings provide the potential of TrkA to be a viable therapeutic target for the treatment of IC/BPS. 

## Authors’ contributions

BSS and SDL designed the experiments; QL, XDL, and JZZ performed experiments and collected data; QL and XDL discussed the results and strategy; BSS and SDL supervised, directed, and managed the study; QL prepared the draft manuscript and visualization. BSS and SDL critically edited the article and approved the final version to be published.

## Conflicts of Interest

The authors declare that they have no conflicts of interest.
